# The Emergence of Saliva as a Diagnostic and Prognostic Tool for Viral Infections

**DOI:** 10.3390/v16111759

**Published:** 2024-11-11

**Authors:** Nilson Ferreira de Oliveira Neto, Rafael Antônio Velôso Caixeta, Rodrigo Melim Zerbinati, Amanda Caroline Zarpellon, Matheus Willian Caetano, Debora Pallos, Roger Junges, André Luiz Ferreira Costa, Juan Aitken-Saavedra, Simone Giannecchini, Paulo Henrique Braz-Silva

**Affiliations:** 1 Department of Stomatology, School of Dentistry, University of São Paulo, São Paulo 05508-000, Brazil; nilsoneto@usp.br (N.F.d.O.N.); rafaelcaixeta@usp.br (R.A.V.C.); amanda.zarpellon@usp.br (A.C.Z.); matheuswillian@usp.br (M.W.C.); 2 Laboratory of Virology (LIM-52-HCFMUSP), Institute of Tropical Medicine, University of São Paulo School of Medicine, São Paulo 05403-000, Brazil; rmzerbinati@usp.br; 3 School of Dentistry, University of Santo Amaro, São Paulo 04743-030, Brazil; dpallos@prof.unisa.br; 4 Institute of Oral Biology, Faculty of Dentistry, University of Oslo, 0316 Oslo, Norway; roger.junges@odont.uio.no; 5 Postgraduate Program in Dentistry, Cruzeiro do Sul University (UNICSUL), São Paulo 1506-000, Brazil; alfcosta@gmail.com; 6 Department of Oral Pathology and Medicine, Faculty of Dentistry, University of Chile, Santiago 3311, Chile; juanpabloaitken@gmail.com; 7 Department of Experimental and Clinical Medicine, University of Florence, 50134 Florence, Italy

**Keywords:** saliva, viral shedding, biomarkers, diagnoses

## Abstract

Saliva has emerged as a promising diagnostic fluid for viral infections, enabling the direct analysis of viral genetic material and the detection of infection markers such as proteins, metabolites, microRNAs, and immunoglobulins. This comprehensive review aimed to explore the use of saliva as a diagnostic tool for viral infections, emphasizing its advantages and limitations. Saliva stands out due to its simplicity and safety in collection, along with the convenience of self-collection without the need for healthcare supervision, while potentially being comparable to urine and blood in terms of effectiveness. Herein, we highlighted the significant potential of saliva in assessing viral loads and diagnosing viral infections, such as herpesviruses, HPV, PyV, TTV, SARS-CoV-2, and MPXV. The detection of viral shedding in saliva underscores its utility in early diagnosis, the monitoring of infection progression, and evaluating treatment responses. The non-invasive nature of saliva collection makes it an appealing alternative to more invasive methods, promoting better patient compliance and facilitating large-scale screening and surveillance. As such, we further highlight current evidence on the use of saliva as a prognostic tool. Although a significant amount of data is already available, further investigations are warranted to more comprehensively assess the added benefit from the utilization of salivary biomarkers in the clinics. Salivary biomarkers show great promise for the early detection and prevention of viral infection complications, potentially improving disease management and control at the population level. Integrating these non-invasive tools into routine clinical practice could enhance personalized healthcare strategies and patient outcomes. Future studies should focus on establishing standardization protocols, validating the accuracy of salivary diagnostics, and expanding clinical research to enhance the diagnostic and monitoring capabilities of salivary biomarkers.

## 1. Introduction

The oral cavity is a diverse and dynamic ecosystem hosting bacteria, fungi, archaea, protozoa, and viruses that constitute the human oral microbiome. The development of a commensal microbiome initiates quickly after birth, developing throughout life [1], leading to an individualized composition likely due to genetic factors coupled with environmental conditions, such as diet and mechanical abrasion, as well as physiologic changes (e.g., pH, salivary composition) [2]. The oral virome consists of both eukaryotic and prokaryotic viruses [3,4,5,6,7]. Bacteriophages, which are prokaryotic viruses, are more prevalent in the human virome than eukaryotic viruses [8,9]. Metagenomic studies consistently identify *Caudovirales* [10,11,12,13] and *Microviridae* [13] as the predominant phage taxa in the healthy oral virome.

Eukaryotic viruses that infect human cells also play a significant role in the human virome. These eukaryotic viruses mainly include *Anelloviridae, Herpesviridae, Papillomaviridae*, *Polyomaviridae* [14,15], and *Redondoviridae* [16,17]. Moreover, some viruses, such as *Orthomyxoviridae* (influenza A) and *Coronaviridae*, e.g., human coronavirus (HCoV) and severe acute respiratory syndrome coronavirus 2 (SARS-CoV-2), can cause recurrently acute respiratory infections, and thus persisting in oral cavity of humans. Herpesviruses can cause acute infections that remit after a few days, with episodes of recurrence and lifelong latency [18,19]. Other viruses, such as the Torqueteno virus (TTV) of the *Anelloviridae* family [20,21,22] and Redondovirus [23,24], colonize the human host without being linked to any disease, suggesting a commensal relationship. Moreover, oncogenic viruses, such as Papillomavirus, can cause infections presenting as warts on various body parts, including the skin, anus, pelvis, and cervix [25,26], with some high-risk genotypes associated with carcinogenesis [25].

Given the diverse composition of the oral virome, it is crucial to consider non-invasive and practical methods for studying and diagnosing these viral populations. Saliva collection is an ideal method in this regard due to its safety, ease of storage, painless nature, speed, and simplicity, requiring minimal handling [27]. Saliva is composed of approximately 98% water and 2% electrolytes, mucus, enzymes, proteins, salivary peptides, mRNA, extracellular RNAs, microRNAs, metabolites, lipids, carbohydrates, and various species of microorganisms, including viruses [28]. This rich composition makes saliva a valuable source of biomarkers, offering a promising alternative for the early diagnosis of systemic diseases and furthering our understanding of the oral virome impact on human health [29]. Therefore, this study provides a comprehensive review of the use of saliva as a diagnostic tool for viral infections.

## 2. Herpesviruses

The viruses of the *Herpesviridae* family are ubiquitous and latent, as they incorporate their genome into the DNA of infected cells after primary infection. Human herpesviruses (HHVs) are enveloped, double-stranded DNA viruses with a linear genome ranging from 120 to 240 kilobase (kb), and they are subdivided into three subfamilies: α-herpesviruses composed of herpes simplex viruses 1 (HSV-1), 2 (HSV-2), and Varicella Zoster (VZV); β-herpesviruses, represented by human herpesviruses 6 (HHV-6A and HHV-6B), human herpesvirus 7 (HHV-7), and cytomegalovirus (CMV); γ-herpesviruses which are represented by Epstein–Barr virus (EBV) and human herpesvirus 8 or Kaposi’s sarcoma-associated herpesvirus (HHV-8 or KSHV) [30].

HHVs are commonly associated with an immunocompromised status, and thus, often associated with the human immunodeficiency virus (HIV). HHVs can cause numerous oral diseases that often recur, persist, provoke serious symptoms, and pose life-threatening risks for people living with HIV/AIDS (PLWHA) [31]. Despite the advent of combined antiretroviral therapy (cART), oral diseases linked to HHV in PLWHA remain prevalent and present ongoing challenges [32]. While antiviral medications have shown promising results in treating active HHV infections, there is currently no approved treatment specifically targeting HHV-6 or EBV infections [33].

Immunocompromised individuals are prone to a recurrence of herpes symptoms due to the latency stage facilitated by these viruses. In such cases, the detection of herpesviruses is typically carried out through blood sample analysis. Notwithstanding, saliva samples exhibit significant potential in identifying initial infection or even preclinical stages [34]. The latency established by HHV occurs following the initial infection, rendering it a lifelong condition. During recurrence periods, viruses may be detectable in saliva even in the absence of symptoms [35].

### 2.1. Alphaherpesvirus

HSV-1 and HSV-2 belong to the Alpha herpesvirus subfamily and have a high prevalence in humans, often manifesting as mucocutaneous lesions in the oral cavity and genital region, respectively [36]. Both viruses are considered highly significant human pathogens with a high transmission rate exhibiting latency in neurons from the peripheral nervous system. HSV-1 is primarily transmitted through saliva, while HSV-2 is transmitted through sexual contact; other routes, such as transmission through small skin lesions, are also reported. The salivary shedding of HSV-1 occurs asymptomatically but also may coincide with the appearance of characteristic lesions such as vesicles or ulcers during primary infection or reactivation. In contrast, HSV-2 causes oral lesions much less frequently compared to HSV-1, resulting in infrequent salivary shedding even in immunosuppressed patients, such as PLWHA [37].

The Varicella Zoster virus (VZV), more recently named and known as human herpesvirus 3, belongs to the same subfamily as the two Herpes simplex viruses, but it has various genetic characteristics that distinguish it from the aforementioned species. VZV persists latently in neurons, and can reactivate in individuals with compromised immune systems, resulting in the development of Herpes Zoster. This can sometimes progress to complex neurological complications. Currently, antiviral medications and vaccines are available to treat this condition [38]. During reactivation, VZV can be detected in saliva, especially in cases of Herpes Zoster. Furthermore, the presence of VZV in saliva is observed in patients with viral meningoencephalitis caused by the virus, highlighting its clinical significance. The detection of VZV DNA in saliva can also serve as a marker for enteric Zoster, indicating an active virus presence in the infection [39].

### 2.2. Betaherpesvirus

Cytomegalovirus (CMV), also known as Human Herpesvirus 5, is a Beta herpesvirus that infects individuals of all ages and is distributed globally. While most infections are asymptomatic, complications can arise during the viral reactivation of virus latently found in monocytes and salivary glands in immunocompromised patients. CMV can also exhibit high levels of shedding in the oral mucosa of PLWHA compared to HIV-negative individuals, potentially increasing CMV transmission through this route. Immunocompromised patients, such as the recipients of solid organ or hematopoietic stem cell transplants, may experience high rates of CMV shedding [40,41].

Human Herpesviruses 6 and 7 are similar viruses belonging to the Beta herpesvirus subfamily. HHV-6 is divided into two species: A and B. While HHV-6A infection is not associated with any disease, HHV-6B and HHV-7 cause infections in children, mainly exanthematous diseases. Following the initial infection, both viruses can establish latency in various sites in the body, such as salivary glands, monocytes, and the central nervous system. The reactivation of these viruses mainly occurs in immunocompromised individuals and is associated with fever, rash, and bone marrow suppression. However, healthy adults may also exhibit the shedding of these viruses in saliva [42].

### 2.3. Gammaherpesvirus

Epstein–Barr virus (EBV), currently known as Human Herpesvirus 4, is considered a lymphotropic virus. It is the most common human herpesvirus, reaching prevalences of up to 90% in the adult population worldwide. The virus can also present latency in B lymphocytes. Classified by the World Health Organization as a Class I oncogenic virus, Epstein–Barr Virus (EBV) is associated with various malignancies, including Burkitt’s lymphoma and Hodgkin’s lymphoma, as well as epithelial malignancies such as nasopharyngeal carcinoma. Additionally, EBV has been linked to autoimmune disorders, including multiple sclerosis and systemic lupus erythematosus [43].

EBV infection can occur in the epithelium of the oral cavity, leading to various local manifestations, such as hairy leukoplakia in immunocompromised individuals [44]. Even asymptomatic individuals may exhibit the lifelong salivary shedding of the virus. EBV is frequently detected in PLWHA and transplant recipients. The salivary shedding of EBV is also common in pediatric patients with chronic kidney disease and renal transplant recipients, with higher rates compared to healthy children [45].

Human Herpesvirus 8, associated with Kaposi sarcoma, also belongs to the Gamma herpesvirus subfamily along with EBV. Like other herpesviruses, HHV-8 is predominantly transmitted through saliva. Viral shedding in saliva is frequent and recurrent in infected individuals, especially in PLWHA, due to the virus’s ability to infect and replicate in oral epithelial cells. However, studies in non-endemic areas for HHV-8 report intermittent viral shedding in saliva [46].

## 3. Human Papillomavirus (HPV)

Human papillomavirus (HPV) is a non-enveloped virus from the *Papillomaviridae* family, featuring a 7.8 kb double-stranded circular DNA genome encased in an icosahedral capsid. HPV is classified based on tissue tropism, infecting either skin or mucous membranes, and further categorized by oncogenic risk as either high or low [47]. Often, the highest oncogenic risk is characterized by subtypes 16 and 18, which present oncogenes shown to affect the functionality of tumor-suppressor proteins, e.g., p53 and Rb, in the host. In the oral cavity, benign HPV-associated lesions (such as leukoplakia, fibrous hyperplasia, papilloma/condyloma, and Heck syndrome) can be observed, caused by types 1, 2, 4, 6, 7, 11, 13, 16, 18, 32, or 57 [48]. HPV is notably implicated in the malignancies of infected tissues, particularly cervical cancer. Globally, HPV is found in approximately 35% of all biopsies of head and neck squamous cell carcinoma lesions, predominantly in the oropharynx, where it is detected in 45% to 90% of cases [49].

HPV-16 is implicated in 40% to 80% of all oropharyngeal cancers in North America [50]. While the detection of various HPV types does not confirm the presence of a cancerous lesion, it aids in patient risk stratification. Studies have explored DNA-HPV detection as a biomarker for early head and neck cancer detection and patient monitoring [51,52]. Most individuals clear HPV infection within two years [53,54]. However, studies have identified persistent HPV-DNA in the saliva of individuals with or previously treated for HPV-associated head and neck cancers, even when asymptomatic [55,56]. One study noted an overall low prevalence of HPV infection, including high-risk genotypes (HPV 16–18) in both urine (8.1%) and saliva (0.7%) samples collected from young adults aged 18–30 years, likely influenced by vaccination strategies modifying infection epidemiology [57].

Saliva has been proposed as a biomarker source for the early and convenient diagnosis/prognosis of head and neck cancer [58], and for monitoring the impact of HPV vaccination strategies. Following the recognition of the role of HPV in various cancers, efforts to reduce infection rates in vulnerable populations have included HPV testing-based screening and vaccination programs. Evidence suggests that vaccination efforts may alter the epidemiology of HPV-related oropharyngeal squamous cell carcinoma, although research on oral HPV infections remains limited [57].

## 4. Torquetenovirus (TTV)

Torquetenovirus (TTV) is a naked small virus belonging to the *Anelloviridae* family, with a single-stranded DNA of approximately 3.8 kb in size. It was first discovered in humans in 1997, in a patient with post-transfusion hepatitis [20]. It is believed that the virus induces chronic or long-term infections, yet without negative effects, meaning that it is not associated with any pathological condition [59]. Moreover, TTV can be considered a pantropic virus, and can be detected in various specimens such as plasma/blood, saliva, and serum [60,61].

Evidence suggests that the replication of TTV in immunocompetent individuals is tightly controlled by the immune system, and that adaptive immune responses play a significant role in controlling its replication, thereby reducing detectable levels [60,62]. Thus, quantifying viral loads in plasma is a potential measure reflecting the functionality of the immune response [59]. Some studies have shown a correlation between disease progression or the worse prognosis of infectious and inflammatory processes with increasing or higher TTV loads [63,64,65].

The oral shedding of TTV has been identified in saliva samples and has been correlated with immunosuppression in conditions such as transplant patients and those immunosuppressed by HIV [66,67,68]. More recently, the oral shedding of TTV has been investigated in patients with COVID-19, as justified by the immune imbalance involved in the immunopathogenesis of SARS-CoV-2 infection, which could influence TTV viral loads [69,70].

Several studies have reported a higher incidence of TTV detection in saliva compared to plasma or blood samples [67,71,72]. This finding highlights the utilization of saliva to monitor one’s individual immune status as promising. The increased viral load of TTV in saliva compared to plasma, observed in both healthy individuals and those with suppressed immune systems, suggests that the oral cavity may be a significant site for viral replication during TTV infection or reactivation. This also indicates that saliva could be a primary transmission route, significantly contributing to the overall viral load [71,72,73].

Our group recently explored the presence of TTV-DNA in the saliva of hospitalized COVID-19 patients and its relationship with disease severity [63]. We found that variables indicative of severe disease, such as the need for intubation and patient unresponsiveness, were associated with TTV-DNA positivity. Notably, TTV-DNA levels were significantly higher in patients with severe disease and those who succumbed to the illness compared to those with moderate disease who were eventually discharged. These findings suggest that TTV-DNA could potentially serve as a biomarker for assessing the severity and prognosis of COVID-19. However, further research is required to validate TTV-DNA as a reliable indicator of clinical disease outcomes.

## 5. Polyomavirus (PyV)

Polyomaviruses are non-enveloped, double-stranded DNA viruses belonging to the *Polyomaviridae* family, with genomes approximately 5 to 7 kilobases in size, causing persistent infections. It is ubiquitous and maintains a symbiotic relationship with both humans and animals. Among the 13 species of Polyomavirus infecting human, the main PyV-associated human diseases include progressive multifocal leukoencephalopathy for human polyomavirus JC (JCPyV), polyomavirus-associated nephropathy for human polyomavirus BK (BKPyV), Merkel cell carcinoma for Merkel Cell Virus (MCPyV), and trichodysplasia spinulosa for trichodysplasia spinulosa-associated polyomavirus (TSPyV) [74]. Primary infection typically occurs during childhood, around 4 to 5 years of age, through respiratory or oral routes, often presenting as subclinical or causing mild respiratory illness [75]. Other potential modes of transmission include the following: (1) airborne transmission via droplets of suspended bodily fluids [76]; (2) fecal–oral transmission [75]; and (3) other routes such as oral–urinary transmission or seroconversion following solid organ transplantation, such as kidney transplants [77]; and (4) through blood transfusion or vertical transmission [78].

In the general population, despite the high prevalence of latent polyomavirus infections or transient asymptomatic activations of latent virus, which range from 20% to over 90% [74], disease manifestation is rare [79]. Among renal transplant patients, the BKPyV is the most common infection [80], with the individual’s degree of immunosuppression being the primary risk factor for disease development [79]. In these patients, BKPyV infection can lead to BK virus-associated nephropathy [81] as well as graft failure in kidney transplant recipients [82].

Increasing evidence has reported an association between human PyVs and the oral compartment [83]. In this regard, JCPyV-DNA and BKPyV-DNA have been detected in respiratory tract secretions, tonsils, and salivary glands [81]. Saliva has demonstrated a prevalence of 91.7% for BKPyV and has been effectively compared to urine and blood tests. Its replication also occurs in glandular cells in vitro. Therefore, saliva can be used for the early detection of BKPyV, and its collection method, whether unstimulated or through rinsing, does not affect laboratory analyses for virus detection. Consequently, the concurrent use of oral fluids alongside other fluids, such as urine and blood, increases positive screening and provides an appealing complementary alternative for polyomavirus detection [81,82].

## 6. Mpox Virus (MPXV)

The Mpox virus (MPXV) is a linear, double-stranded DNA virus enclosed in a lipoprotein envelope, with a genome size of approximately 197 kb. It belongs to the *Poxviridae* family, which comprises two clades: Clade I (formerly known as the Congo Basin clade) and Clade II (formerly known as the West African clade), with Clade II further subdivided into subclades IIa and IIb. In 2024, a new subclade of Clade I, named Clade Ib, was identified in the African region [84].

In early July 2022, MPXV rapidly spread to multiple non-endemic countries, prompting an emergency alert from the World Health Organization (WHO), which designated it as a Public Health Emergency of International Concern (PHEIC). The WHO declared it a global outbreak, noting documented community transmission among individuals who had contact with symptomatic cases in non-endemic countries. However, on 11 May 2023, the Emergency Committee on Mpox reported that the outbreak was no longer considered a PHEIC due to a considerable decrease in the number of cases [85]. Nevertheless, due to the emergence of the new Clade Ib and the resurgence of cases in the African region, a PHEIC was once again declared on 14 August 2024 [84].

According to the Center for Disease Control and Prevention (CDC), the transmission occurs through direct contact with infected skin lesions, oral fluids, respiratory tract secretions, or contaminated fomites like objects and clothing. Several unconventional factors are associated with the outbreak; for instance, there is a higher prevalence of cases among men who have sex with men (MSM), though not exclusively [86]. Moreover, typical disease manifestations, such as skin lesions on the face and extremities, have now expanded to include genital and perianal regions, with or without dissemination to other areas [87]. This suggests that intimate contact with these lesions during sexual activity is a probable mode of transmission. However, MPXV does not yet fit within the group of sexually transmitted infections (STIs) as traditionally described [88,89].

It is believed that immunopathogenesis directly influences the severity and progression of the disease, as well as the outcome of infections caused by viruses of the *Orthopoxvirus* genus. Recent studies have reported that the progression of MPXV may be linked to elevated serum concentrations of IL-4, IL-10, GM-CSF, and Sil-2R, alongside moderate expression of IL-2, IFN-γ, and IL-12. This cytokine profile promotes an environment conducive to the rapid dissemination and progression of infection, akin to what is often observed in HIV and hepatitis C virus (HCV) infections [90].

In recent studies during the 2022/2023 outbreak, MPXV DNA was present in all saliva samples collected between 4 and 16 days [91] and 76 days [92] from symptom onset. Allan-Blitz and collaborators have shown that 88.9% of MPXV-infected patients tested positive through both saliva-based analysis and skin lesion swabs [93]. Another study demonstrated the agreement between saliva and skin tests for MPXV DNA detection, which showed 93.5% sensitivity to mpox salivary diagnostics. Additionally, the use of reverse transcription polymerase chain reaction (RT-PCR) has shown increased salivary loads of MPXV DNA, and another study revealed that infectious MPXV virions were found in 67.5% of saliva samples from subjects with MPXV [94]. Hence, saliva represents a valuable tool for asymptomatic screening, enabling early interventions and reducing transmission. Future studies should be conducted to further elucidate the dynamics of MPXV shedding, particularly in light of the newly recognized transmission route involving saliva [95].

## 7. Arboviruses

Arthropod-borne viruses (arboviruses) have rapidly increased globally due to urbanization and climate change, becoming a significant public health issue and causing disease outbreaks. Considerable morbidity and mortality are associated with the four serotypes of dengue viruses (DENV1-4), resulting in nearly 500 million infections each year. Additionally, the emergence of chikungunya (CHIKV) and Zika virus (ZIKV) in areas with high dengue prevalence is of significant concern [96].

DENV and ZIKV belong to the *Flaviviridae* family, both classified as enveloped, single-stranded positive-sense RNA viruses with a genome size of approximately 11 kb. Similarly, CHIKV is a positive-sense single-stranded RNA virus, but it belongs to the *Togaviridae* family and has a slightly larger genome, measuring around 12 kb. These viruses are interconnected in urban environments, sharing humans as common hosts and primarily using *Aedes aegypti* as their vector. Their overlapping geographic distribution, infection rates, and parallel seasonality have led to simultaneous outbreaks in various global regions. Besides DENV, ZIKV, and CHKV, other viruses such as yellow fever, West Nile, Mayaro, and Japanese encephalitis also co-circulate in urban and surrounding areas [97].

DENV is the most prevalent urban arbovirus in the Americas. Its occurrence is widespread, mainly affecting tropical and subtropical countries where climatic and environmental conditions favor the development and proliferation of the vectors [98]. On the other hand, an increase in cases of severe febrile illness has been observed among individuals diagnosed with the alphavirus CHIKV, due to alterations in the dynamics of infection. Additional arboviruses have been studied outside of these families, including tick- and sandfly-borne viruses found in the extensive *Bunyaviridae* family [99].

ZIKV infection is known to be associated with adverse fetal brain outcomes, such as microcephaly and Guillain–Barré syndrome, particularly when the infection occurs during pregnancy [100]. The virus has been identified in breast milk [101], blood, urine, semen [102], and saliva [103], during the acute phase of ZIKV infection in humans, using RT-PCR for detection. Diagnosis is typically performed via RT-PCR to detect viral nucleic acid within seven days of symptom onset or through immunoassays to detect specific IgM antibodies [104].

In contrast, CHIKV can cause arthralgia, arthritis, myalgia, and a febrile illness known as chikungunya fever. According to the European Centre for Disease Prevention and Control (ECDC), approximately 400,000 cases of CHIKV infection have been reported worldwide, predominantly in Brazil, India, Paraguay, Guatemala, and Thailand [105]. Some studies have identified the presence of CHIKV in saliva. One study confirmed that viral RNA was detected in approximately 60% of saliva samples during the first week after the onset of symptoms [106]. Additionally, another study involving several acute CHIKV patients with hemorrhagic manifestations demonstrated the presence of both viral RNA and infectious virus in saliva samples [107].

Oliveira and collaborators have demonstrated the potential of using salivary infrared signatures for high-accuracy detection in an animal model ZIKV infection. They utilized a reagent-free Attenuated Total Reflection Fourier Transform Infrared Spectroscopy (ATR-FTIR) platform, which is commonly employed for biofluid analysis [108]. Similarly, the same research group developed an animal model for CHIKV, and the results indicated that salivary infrared signatures can effectively detect the virus using the same sustainable biophotonic platform. Specifically, the findings showed a sensitivity of 83%, a specificity of 86%, and an overall accuracy of 85% when employing support vector machine algorithms on the infrared signatures of saliva samples from CHIKV-infected and control mice [109]. This method enables same-day detection of both viruses, creating new opportunities for monitoring and diagnosing a range of diseases.

## 8. Redondovirus (RdVs)

In 2019, a new family of small, circular DNA viruses called *Redondoviridae* was identified, predominantly found in human lung and oropharyngeal samples. The RdVs genome consists of circular single-stranded DNA (ssDNA), measuring approximately 3 kb in length and containing three genes. Two of these genes encode a capsid protein and a replication-associated protein, while the third, ORF3, encodes a protein of unknown function. RdVs exhibit a global distribution, having been detected in studies across various countries. They are commonly found in the respiratory tract of both healthy and diseased individuals, with prevalence rates ranging from 2% to 82% [110,111]. Furthermore, the virus has been identified in the human oral cavity, including in critically ill patients and those with periodontitis [16].

Periodontitis is a progressive inflammatory disease triggered by a variety of factors including a dysbiotic microbiome. RdVs sequences have been linked to periodontal disease, with their levels decreasing after treatment. It is hypothesized that RdVs infection and replication may help maintain the inflammatory state associated with periodontitis, thereby promoting disease progression. The prevalence of RdVs appears to occur in both healthy individuals and critically ill patients, although levels are higher during illness. Additionally, the upper and lower respiratory tracts seem to serve as stable niches for redondovirus detection over time [16]. Thus, the role of RdVs in periodontitis warrants further study.

RdVs are hypothesized to replicate either in human cells or in the eukaryotic parasite *Entamoeba gingivalis*, a potential viral host found in human saliva. *Entamoeba gingivalis* DNA was detected in 46% of 28 saliva samples, and a strong correlation between RdVs DNA and *E. gingivalis* DNA was observed in 93% of these cases. These findings, combined with previous studies, strongly support the presence of RdVs in the human oral cavity and suggest that *E. gingivalis* is likely their host [112].

## 9. Respiratory Viruses

The use of saliva as a diagnostic tool for seasonal respiratory viruses has been widely discussed, especially after the COVID-19 pandemic, due to the successful development and implementation of saliva-based diagnostic tests during that time period. Current investigations focused on comparing the efficacy of different diagnostic samples concluded that saliva and nasopharyngeal specimens (NSs) were highly consistent for detecting respiratory tract infection pathogens such as adenovirus, rhinovirus, enterovirus, human metapneumovirus, parainfluenza virus, bocavirus, and human coronaviruses. Moreover, salivary gp-340, histatins, MUC5B, and human neutrophil defensins in saliva have been shown to neutralize influenza A virus [113,114,115].

Although the use of saliva to detect respiratory viruses is rare, as it is commonly considered to have inferior sensitivity compared with other fluids, the interest in using saliva for detecting respiratory viruses through molecular assays has been revisited. Some studies demonstrated that the detection rate of respiratory viruses was actually superior for saliva compared to NS using RT-PCR [116,117]. One cohort study using multiplex PCR showed that using both collection methods enhanced the clinical management of patients with suspected respiratory infections [118]. Kim et al. showed a similar result and concluded that saliva and NS had similar detection rates using multiplex PCR [119].

There are numerous advantages to collecting saliva instead of NS. The first and main advantage is that the use of saliva is more comfortable for the patient. Secondly, when the collection of nasopharyngeal specimens is not recommended (e.g., patients at high risk for hemorrhagic events), the collection of saliva is a safer option. Thirdly, following standard protocols, the collection of saliva can be performed in any clinical setting. The methods for nasopharyngeal collection are potentially aerosol-generating and consequently pose considerable risks to healthcare workers and patients. Fourthly, the time and cost associated with the use of saliva are lower than those for using nasopharyngeal specimens. Lastly, this method allows for better adherence in clinical studies and self-collection in community surveillance studies [120].

## 10. SARS-CoV-2

Coronaviruses are a family of enveloped, positive-sense, single-stranded RNA viruses that infect both avian and mammalian species. Their single, linear RNA genome ranges in size from 26 to 32 kb, making it the largest known RNA virus genome [121]. Four seasonally circulating coronaviruses are documented: HCoV 229E, HCoV-OC43, HCoV-NL63, and HCoV-HKU1. These typically cause mild common cold symptoms in humans. In contrast, severe acute respiratory syndrome coronavirus (SARS-CoV), Middle East respiratory syndrome coronavirus (MERS-CoV), and SARS-CoV-2 emerged in 2002, 2012, and 2019, respectively [122]. Unlike the milder strains, these viruses can cause more severe respiratory diseases. They belong to the *Coronaviridae* family, within the *Nidovirales* order. Since it was first identified in Wuhan in December 2019, the virus that causes COVID-19, SARS-CoV-2, has spread across the globe [123].

The infection of host cells by SARS-CoV-2 depends on the cleavage of one of its spike protein subunits by the enzyme furin. This cleavage event facilitates the binding of the cleaved spike protein to the angiotensin-converting enzyme 2 (ACE2) receptors and the transmembrane serine protease 2 (TMPRSS2). These crucial interactions trigger cellular endocytosis, marking the beginning of the viral replication cycle [124]. Evidence from animal studies shows the interaction between these receptors and SARS-CoV-2, indicating that ACE2, TMPRSS2, and furin in the tissues of the salivary glands are among the first targets of coronavirus infection [125].

SARS-CoV-2 can be detected in saliva as its composition includes secretions from the salivary glands, crevicular fluid, secretions from the lower and upper respiratory tracts, and desquamated epithelial cells. Therefore, the presence of the virus in the oral cavity may be related to different sources. Through the use of various approaches, including immunohistochemistry, biomolecular analysis, and histology combined with electron microscopy images, it was observed that both major and minor salivary glands play a role in harboring the SARS-CoV-2 virus and facilitating its replication process. Ultrastructural analyses demonstrated viral particles in the cytoplasm of duct-lining cells, acinar cells, and in the ductal lumen of the salivary glands [126]. These findings emphasize the role of salivary glands as a reservoir for SARS-CoV-2, providing a fundamental physiological context to complement recent research suggesting the utility of saliva as a diagnostic tool for COVID-19.

There is already evidence demonstrating the presence of SARS-CoV-2 RNA in periodontal tissues. The detection of viral RNA persisted in these tissues for a considerable duration after the initial symptoms in the examined patients [124]. These findings support the role of the oral cavity as a reservoir for SARS-CoV-2, given its consistent detection in saliva [127]. This also suggests a possible connection with the presence of the virus through specific routes of access to crevicular fluid and emphasizes the significant contribution of this biological fluid to the transmission of the disease.

Given that a coronavirus depends on the ACE2 as a receptor to invade human cells, it is conceivable that cells expressing this receptor may be susceptible to SARS-CoV-2 infection. This could lead to an inflammatory response in related organs and tissues, including the tongue mucosa, oral mucosa, and salivary glands, and could also result in changes in taste responses. Indeed, the findings from observations of oral manifestations in COVID-19 patients, documented in various studies, support the notion that such oral lesions are secondary and result from coinfection and immune suppression, due to either treatment or the disease itself [127,128,129].

Studies using minimally invasive autopsy techniques have documented a series of cases that included the presence of vesicular-bullous and ulcerative lesions, indicative of herpes simplex virus infection, as well as erythematous lesions associated with *Candida* spp. coinfection in fatal cases of COVID-19. Additionally, extensive ulcerative lesions on the lips, apparently resulting from trauma caused by prolonged orotracheal intubation in severe patients, were identified, highlighting the role of dental surgeons in the diagnostic process. These instances suggest that these oral manifestations potentially arise as adverse responses to therapeutic and supportive interventions for COVID-19 [130].

Furthermore, regarding oral manifestations related to taste alterations, the presence of SARS-CoV-2 virus replication was confirmed through in situ hybridization in type II cells, a subset of taste receptor cells located in taste buds. These findings demonstrate that taste alterations, often observed in COVID-19 patients, may arise from the direct infection of taste buds by the SARS-CoV-2 virus [130].

## 11. Final Considerations

This review has highlighted the significant potential of saliva in the diagnostic of viral infections, such as herpesviruses, HPV, TTV, PyV, SARS-CoV-2, Arbovirus, and MPXV. A summary of our findings is presented in Table 1. The detection of viral shedding in saliva underscores its utility in early diagnosis, the monitoring of infection progression, and in the evaluation of treatment responses. The non-invasive nature of saliva collection makes it an appealing alternative to more invasive methods, promoting better patient compliance and facilitating large-scale screening and surveillance. In highly contagious viral diseases, the development of home-use salivary testing devices could allow patients to perform sample analysis from home, reducing the risk of viral transmission. However, several considerations should be noted. Saliva testing, influenced by factors such as age, gender, and diet, has some limitations, including the following: (i) the potential levels of biomarkers that may not fully represent the levels of those present in serum; (ii) the impact of salivary flow and collection methods on biomarker composition; (iii) the influence of external factors, such as the consumption of medications, food, and beverages, on saliva composition; and (iv) the stability of biomarkers in saliva, which can be affected by proteolytic enzymes. In addition, the saliva biomarker response is also affected by the collection method including unstimulated or stimulated whole saliva, unstimulated or stimulated sublingual/submandibular saliva, and unstimulated or stimulated parotid saliva.

In light of the COVID-19 pandemic, recent scientific evidence indicates that molecular tests performed on saliva can have diagnostic sensitivity and specificity comparable to, or even superior to, those observed with nasopharyngeal swabs. However, the collection and processing of saliva are often poorly standardized, which can negatively impact SARS-CoV-2 detection. It is important to note that saliva samples can originate from different sites, including posterior oropharyngeal saliva and oral saliva, each with distinct characteristics. Furthermore, challenges related to the viscosity of saliva represent another potential limitation in the use of this matrix for diagnostic purposes, necessitating improved collection and processing methods to enhance reliability. Therefore, future studies should focus on establishing standardization protocols, validating the accuracy of salivary diagnostics, and expanding clinical research to enhance the diagnostic and monitoring capabilities of salivary biomarkers. Moreover, while the microbiome field at large has been mostly concentrated in bacteria, the further exploration of the interplay between the human bacteriome and virome during health and disease is warranted and will likely expand the array of diagnostic possibilities. By advancing our understanding and application of salivary diagnostics, we can provide a powerful and convenient tool for clinical practice, significantly impacting public health and disease management. This review underscores the importance of expanding research and development in this promising field, highlighting the diagnostic potential of saliva leading to an improvement in healthcare outcomes.

## Figures and Tables

**Table 1:** Salivary shedding and detection of viruses: family, subfamily, genetic material, and clinical relevance.

Virus	Subfamily	Family	Genetic Material	Size	Detection in Saliva	References
**HSV1**	α-herpesviruses	*Herpesviridae*	Double-stranded linear DNA	152 kb	Salivary shedding of HSV-1 can occur asymptomatically but may also coincide with the appearance of characteristic lesions such as vesicles or ulcers during primary infection or reactivation.	Tognarelli et al., 2019 [36]
**VZV**	α-herpesviruses	*Herpesviridae*	Double-stranded linear DNA	125 kb	It is possible to detect it in cases of Herpes Zoster, viral meningoencephalitis, and enteric zoster.	Gershon et al., 2015 [39]
**CMV**	β-herpesviruses	*Herpesviridae*	Double-stranded linear DNA	235 kb	Salivary CMV shedding is common in immunocompromised individuals, such as transplant recipients and PLWHA, who often exhibit higher shedding rates.	Riis et al., 2020 [41]
**EBV**	γ-herpesviruses	*Herpesviridae*	Double-stranded linear DNA	172 kb	EBV is associated with hairy leukoplakia in immunocompromised individuals, with lifelong salivary shedding observed in asymptomatic individuals, particularly in PLWHA, transplant recipients, and pediatric patients with chronic kidney disease or renal transplants.	Rosseto et al., 2023 [44]; Sarmento et al., 2020 [45]
**HHV-8**	γ-herpesviruses	*Herpesviridae*	Double-stranded linear DNA	165 kb	Viral shedding in saliva is frequent and recurrent, especially in PLWHA, due to the virus’ ability to infect and replicate in oral epithelial cells, though studies in non-endemic areas report intermittent shedding for HHV-8.	Braz-Silva et al., 2017 [46]
**HPV**	*Papillomavirinae*	*Papillomaviridae*	Double-stranded circular DNA	7.8 kb	Saliva is proposed as a biomarker for early diagnosis, prognosis of head and neck cancer, and monitoring the impact of HPV vaccination strategies.	Ekanayake et al., 2021 [52]; Tang et al., 2020 [55];
**TTV**	*Anellovirinae*	*Anelloviridae*	Single-stranded circular DNA	3.8 kb	Oral shedding of TTV in saliva is correlated with immunosuppression, and recent studies suggest that saliva may serve as a key site for the viral replication, transmission, and a potential biomarker for disease severity, including in COVID-19 patients.	Caixeta et al., 2024 [63]
**PyV**	*Polyomavirinae*	*Polyomaviridae*	Double-stranded circular DNA	7 kb	Saliva has been identified as a viable medium for the early detection of human polyomaviruses, particularly BKPyV, which shows a high prevalence in oral samples and allows for effective screening alongside urine and blood tests.	Sarmento et al., 2019 [82]
**MPXV**	*Chalmydovirinae*	*Poxviridae*	Double-stranded linear DNA	197 kb	Recent studies from the 2022/2023 MPXV outbreak show that MPXV DNA is present in saliva for up to 76 days post-symptom onset, with high diagnostic sensitivity (93.5%) and the virus found in 67.5% of samples.	Peiró-Mestres et al., 2022 [91]; Allan-Blitz et al., 2023 [93]; Hernaez et al., 2023 [94]
**ZIKV**	*Flavivirus*	*Flaviviridae*	Single-stranded RNA	11 kb	The use of salivary infrared signatures for high-accuracy detection of ZIKV infection was demonstrated in an animal model using a reagent-free ATR-FTIR platform.	Oliveira et al., 2023 [108]
**CHIKV**	*Alphavirus*	*Togaviridae*	Single-stranded RNA	12 kb	Studies identified CHIKV RNA in saliva, with 60% of samples testing positive within the first week of symptoms, and a study showed that salivary infrared signatures effectively detected CHIKV with 83% sensitivity, 86% specificity, and 85% accuracy in an animal model.	Musso et al., 2016 [106]; Guevara-Veja et al., 2024 [109]
**RdVs**	*Redondovirinae*	*Redondoviridae*	Single-stranded circular DNA	3 kb	Redondoviruses, linked to periodontitis and present in both healthy and critically ill individuals, are hypothesized to replicate in *Entamoeba gingivalis* found in human saliva, with strong correlation between their DNA and that of the bacteria.	Keeler et al., 2023 [112]
**SARS-CoV-2**	*Orthocoronavirinae*	*Coronaviridae*	Single-stranded linear RNA	30 kb	SARS-CoV-2 has been detected in saliva and periodontal tissues, with salivary glands acting as reservoirs, and its presence in oral mucosa may contribute to oral manifestations such as taste alterations and lesions, potentially due to viral replication or secondary infections.	Matuck et al., 2021 [126]; Braz-Silva et al., 2021 [121]; Zarpellon et al., 2022 [127]
